# Pathologically altered articular cartilage attracts intense chondrocyte invasion into the extracellular matrix: in vitro pilot study

**DOI:** 10.1186/s43019-024-00249-y

**Published:** 2024-12-03

**Authors:** Victoria A. Shestakova, Ilya D. Klabukov, Ilya V. Kolobaev, Longfeng Rao, Dmitry A. Atiakshin, Michael A. Ignatyuk, Mikhail E. Krasheninnikov, Bagavdin G. Ahmedov, Sergey A. Ivanov, Peter V. Shegay, Andrey D. Kaprin, Denis S. Baranovskii

**Affiliations:** 1 National Medical Research Radiological Center of the Ministry of Health of the Russian Federation, Koroleva st. 4, 249036 Obninsk, Russia; 2 Obninsk Institute for Nuclear Power Engineering of the National Research Nuclear University MEPhI, Obninsk, Russia; 3 Patrice Lumumba Peoples Friendship University of Russia (RUDN University), Moscow, Russia; 4 ETH Zurich, Zurich, Switzerland; 5 National Medical Research Center for Surgery named after A.V. Vishnevsky of the Ministry of Health of the Russian Federation, Moscow, Russia; 6 University of Basel, Basel, Switzerland; 7 FSBEI HE “Rosunimed” of MOH of Russia, Moscow, Russia

**Keywords:** Cartilage, Cell therapy, Chondrocytes, Pathologically altered cartilage, Regenerative medicine

## Abstract

**Background:**

Due to non-vascularized and aneural structure, articular cartilage has limited self-repairing capacity. The aim of this study was to investigate the revitalization of inflammatory injured articular cartilage matrices by human nasal chondrocytes (hNC).

**Materials and methods:**

Cartilage matrix was prepared by devitalization of articular cartilage samples obtained intraoperatively from an adult patient undergoing knee joint replacement. hNC were obtained from native tissues by enzymatic digestion with further expansion over two passages. The obtained nasal chondrocytes were used to seed decellularized scaffolds, which were then cultured in vitro for 7, 14, or 21 days in chondrogenic medium. Migration was observed by histologic staining with fast green, safranin-O, and hematoxylin and scanning electron microscopy. Biochemical analysis was performed to determine the glycosaminoglycan (GAG) and DNA content of the cartilage using dimethylmethylene blue and CyQuant Cell Proliferation Assay Kit.

**Results:**

We seeded healthy and inflamed cartilage with nasal chondrocytes and found that the cells actively invade mainly pathologically altered cartilage. The results of biochemical quantitative analysis showed that the amount of DNA significantly increased by day 7 and decreased by day 14, while the quantitative values of GAGs had the opposite trend. Histological staining showed that cartilage formation occurred on day 7, intercellular spaces were filled with de novo synthesized cartilage matrix with significantly low GAG content on day 14, and newly formed GAG-rich cartilage was observed on day 21. The obtained data on cartilage regeneration were confirmed by scanning electron microscopy.

**Conclusions:**

Our preliminary results showed that human nasal chondrocytes are capable of infiltrating the pathologically altered extracellular matrix of articular cartilage damaged by arthritis, thereby promoting its repair to a physiologically relevant state.

**Graphical Abstract:**

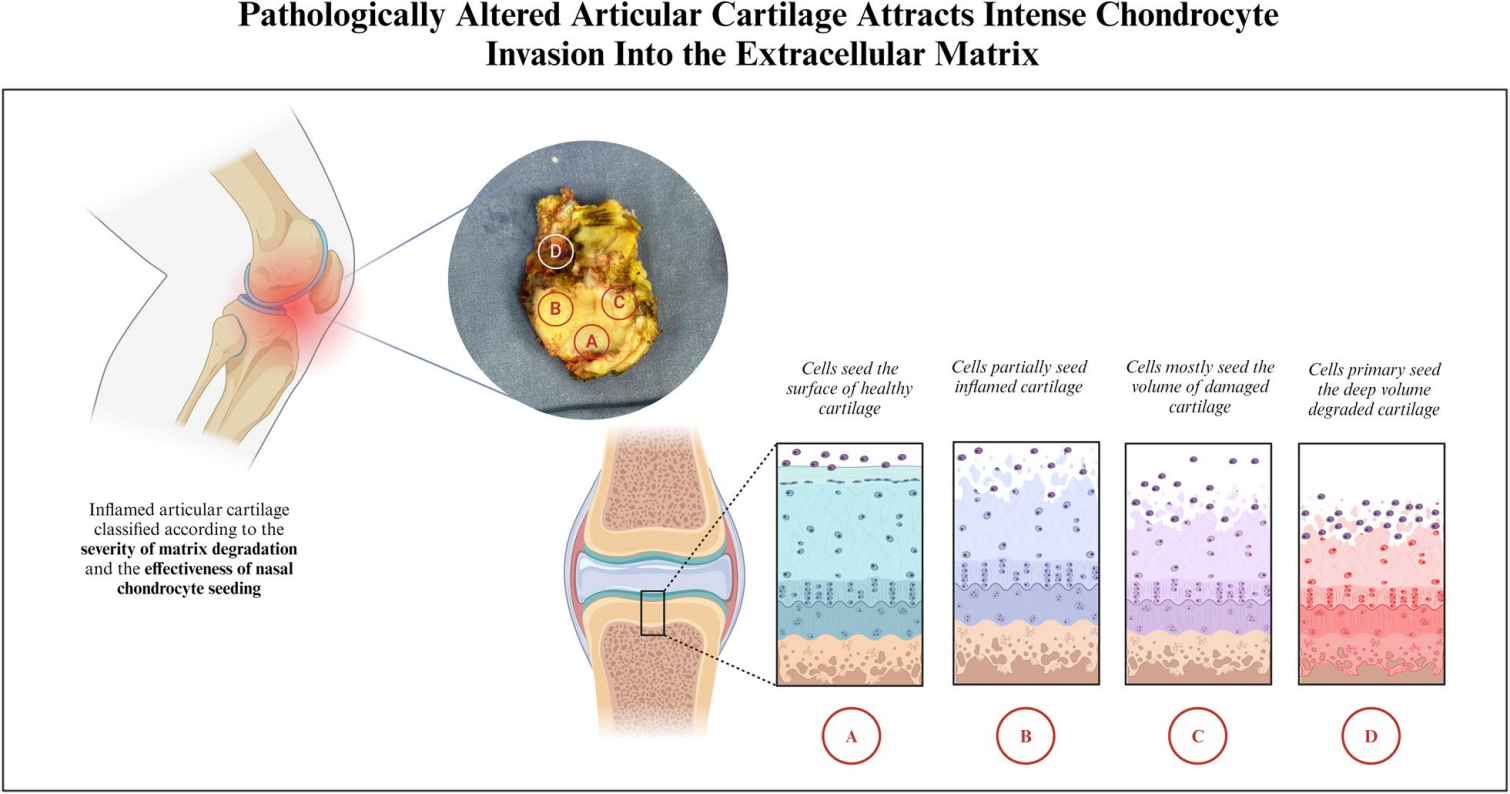

## Background

Cartilage disorders remain a highlighted field for searching for novel regenerative therapeutic approaches, cell sources, and protocols of cell processing [1]. Nowadays, cell-based therapy for treatment of chronic cartilage damage has been widely investigated, especially mesenchymal stem cell (MSC) therapy [2, 3]. The main efficacy of MSC therapy is related to their immunomodulatory and anti-inflammatory properties, which create a pro-regenerative microenvironment or exert a protective effect, ultimately effectively stimulating in situ regeneration [4–6]. The most recent studies have reported adverse events and side effects associated with MSCs therapy, which include: cell transformation in culture, heterogeneity of MSCs, lack of exceptional chondrogenic differentiation capacity, systemic depletion and impaired MSC function [7–9], hypertrophy during differentiation [10], tumor occurrence, and the possibility of chromosomal aberrations and genetic instability in long-term cultured MSCs [11]. The therapy is effective in the treatment of early OA or mild to moderate OA [12]. These findings underscore the need for caution and further research to ensure the safety and efficacy of MSC therapy for OA treatment.

For the last decade, clinicians mainly used autologous chondrocyte implantation (ACI) as the gold standard cell therapy for cartilage restoration. ACI is based on articular chondrocytes expanded in vitro and reimplanted into the cartilage defect [6]. However, the application of ACI has several limitations including deep subchondral bone lesions (more than 8 mm) [13], limited availability of donor sites, small amount of isolated cells, long cell expansion time (2.6–5 ml of cells are obtained in 2 weeks), long cartilage repair time, uncertain long-term clinical efficacy [13–17], ACI administration is currently limited to the treatment of cartilage defects rather than osteoarthritis [12], and the ability to de-differentiate to a fibroblast-like phenotype during culturing [18].

However, these deficiencies apply specifically to articular chondrocytes. Obtaining biological material from nasal cartilage is known to be a less invasive operation than articular cartilage sampling, resulting in fewer postoperative complications and lower morbidity [19, 20]. In addition, the studies, including some recent ones, have shown a higher proliferative and chondrogenic capacity of nasal chondrocytes compared with articular chondrocytes [21, 22]. Nasal chondrocytes manufacture more extracellular matrix (ECM) with rapid cartilage maturation, due to higher expression levels of chondrogenic genes [22, 23]. Matrix contains enhanced compounds of glycosaminoglycans and type II collagen [21–26]. Nasal cartilage is less affected by inflammatory cytokines or acidic conditions than cartilage generated by ACs or differentiated MSCs [27]. Maintaining cartilaginous properties under inflammatory conditions is crucial of osteoarthritic joints, partially mediated by downregulation of the WNT signaling pathway, and is effective in treating progressive OA [28]. That is, as a more advantageous source for cartilage repair and regeneration, nasal chondrocytes can be considered as a preferable source for cartilage engineering [29]. In this regard, we aimed to investigate the revitalization of inflammatory injured articular cartilaginous matrices by human nasal chondrocytes (hNC). We hypothesize that the ECM of inflamed cartilage attracts nasal chondrocytes for enhanced invasion compared to the matrix of healthy cartilage.

## Materials and methods

### Harvesting of human articular cartilaginous matrix

Human articular cartilage was harvested intraoperatively from an adult patient undergoing knee joint replacement. Cartilage samples were isolated from different areas on the tibial surface. Cartilaginous matrix was obtained by devitalization of the samples and continuous washing in cold phosphate-buffered saline (PBS) for 8 h at 4 °C. The samples were devitalized using the freeze and thaw method [30]. Briefly, the tissues were placed into vials that were immersed in liquid nitrogen for 15 min. After that, samples were then thawed in a water-bath at 37 °C for 30 min. This cycle was repeated five times. An equal fragment of each devitalized sample was donated for primary histological analysis. All the samples were then divided into four different groups with five samples in each group, according to the severity of matrix degradation in the sampling area:

A: Normal healthy cartilaginous matrix.
B: Cartilaginous matrix superficially injured by chronic inflammation.
C: Severe deep injury of the matrix with a loss of proteoglycan content.
D: Evidence of cartilage failure followed by a completely disorganized matrix.

Separated matrices were chopped into small fragments (2 mm × 2 mm).

### Nasal chondrocytes

Nasal septum biopsies were received from four patients (female, 25–54 years old) undergoing rhinoplasty. The required sample size was estimated as (*N* + 1), where *N* is the minimum number necessary for statistical analysis (*N* = 3). hNCs were obtained from native tissues via enzymatic digestion (22 h in 1.5 mg/ml collagenase) as previously described by Power et al. [31]. Isolated cells were then expanded in expansion medium [Dulbecco’s modified Eagle medium (DMEM) containing 10% fetal bovine serum (FBS), 10 mM HEPES buffer (Gibco), 1 mM sodium pyruvate (Gibco), 100 U/ml penicillin, 100 µg/ml streptomycin, 0.29 mg/ml l-glutamine (Invitrogen), 1 ng/ml TGF-β1 and 5 ng/ml FGF-2 (both from R&D Systems)] for two passages.

### Revitalization of human articular cartilaginous matrix

Cartilaginous fragments were placed separately onto 0.4 mm pore size polycarbonate Transwell filters (Corning B.V. Life Sciences, Schiphol-Rijk, The Netherlands, two samples/insert). hNCs were applied on the top of the matrix in concentrated cell suspension (100 μl of cell suspension containing 0.5 × 106 cells per insert). Matrices were cultured in vitro for 7, 14 or 21 days in chondrogenic medium [DMEM containing 5% FBS, 10 mM HEPES, 1 mM sodium pyruvate, 100 U/ml penicillin, 100 µg/ml streptomycin, 0.29 mg/ml l-glutamine, 10 µg/ml insulin (Novo Nordisk, Bagsvaerd, Denmark), 0.1 mM ascorbic acid 2-phosphate (Sigma-Aldrich)] at 37 °C and 5% CO_2_ with medium change on each third day. Revitalized cartilaginous matrices were examined histologically or biochemically as described below.

### Histological examination

All samples were fixed in 4% paraformaldehyde for 24 h at room temperature followed by dehydration in rising ethanol concentrations and embedding in paraffin. Paraffin blocks were cut into 5-μm thick serial sections with the microtome (Thermo Fisher Scientific, USA). Slices were placed on uncovered slides. Then the sections were stuck at +56 °C for 30 min. The obtained slides were dewaxed and stained consistently with fast-green, safranin-O and hematoxylin according to standard protocols. Histological images were acquired with light microscopy (Eclipse Ti2, Nikon Corp., Tokyo, Japan). Quantification of ingrowing hNC was accessed by counting alive cell nuclei below the matrix surface.

### Biochemical examination

Biochemical analysis was performed by quantitative measurement of glycosaminoglycans (GAGs) and deoxyribonucleic acid (DNA). Standard protocols were used for this purpose. The quantitative content of glycosaminoglycans (GAGs) was determined spectrophotometrically using dimethylmethylene blue with standard chondroitin sulfate [32]. Briefly, digested pellets were incubated with 1 ml of dimethylmethylene blue assay (DMMB) solution (16 mg/l dimethylmethylene blue, 6 mM sodium formate, 200 mM GuHCL, pH 3.0) on a shaker at room temperature for 30 min. Precipitated DMMB–GAG complexes were centrifuged and supernatants were discarded. Complexes were dissolved in decomplexion solution (4 M GuHCL, 50 mM Na-Acetate, 10% Propan-1-ol, pH 6.8) at 60 °C. The absorption was measured at 656 nm, and GAG concentrations were determined by utilizing a standard curve prepared with purified bovine chondroitin sulfate [33]. Quantitative DNA content was measured using the CyQuant Cell Proliferation Assay Kit (Molecular Probes Inc., Eugene, OR) according to the manufacturer’s instructions.

### Dynamic migration of chondrocytes

Dynamic chondrocyte migration over time was assessed by histological analyses of fixed samples and laser scanning confocal microscopy for live time observation. For histological evaluation, as mentioned previously, standard protocols using fast green, safranin-O and hematoxylin triple staining were used. A devitalized specimen without chondrocytes was used as a control. Serial sections were made both for the control sample and on days 1, 7, and 14 after cell engraftment. Laser scanning confocal microscopy was used to track the dynamics of cell migration over time. For this purpose, cells were labeled with the PKH-26 membrane marker according to the standard protocol [34].

### Scanning electron microscopy

Surface colonization of the samples by nasal chondrocytes was evaluated by scanning electron microscopy with gold sputtering (Nova NanoSEM 230, FEI, USA).

### Statistics

Statistical analysis of the obtained data was carried out in GraphPad Prism 7.0 software (USA) using ANOVA assay. The level of statistical significance was taken as a *p* value < 0.05.

## Results

### Evaluation of human nasal chondrocyte migration on articular cartilage matrices

**Fig. 1:**
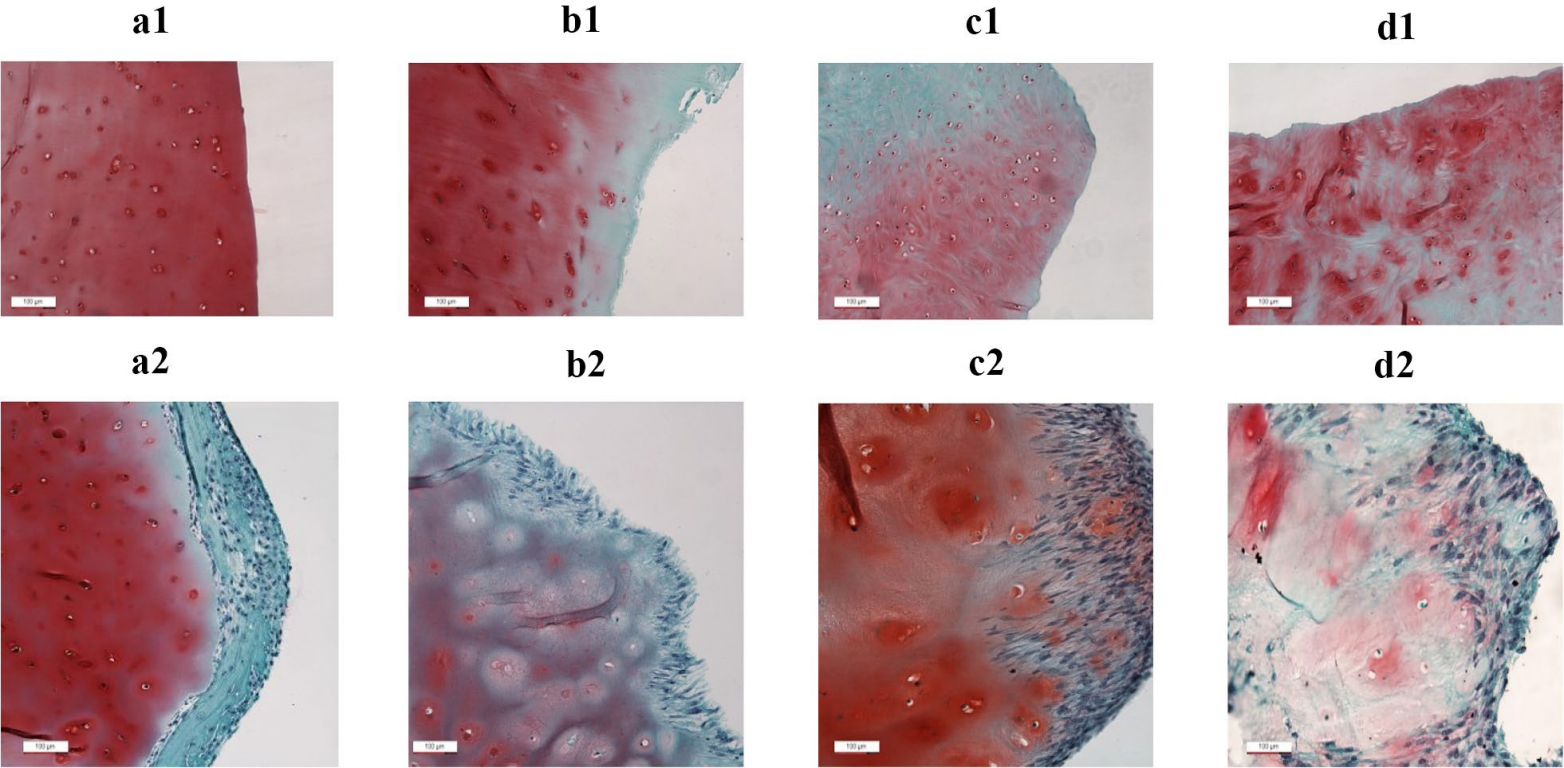
Human knee articular cartilaginous matrix, staining with safranin-O, light microscopy; top row, non-revitalized control fragments; bottom row, fragments revitalized with hNCs; **A** normal healthy cartilaginous matrix; **B** cartilaginous matrix superficially injured by chronic inflammation; **C** severe deep injured matrix with a loss of proteoglycan content; **D** completely disorganized cartilaginous matrix

hNCs were cultured for 1 week on the surface of the articular cartilaginous matrix to access cell ingrowth and repopulation in cartilage-injured locations. Histological analyses found that hNCs cannot penetrate the healthy cartilaginous matrix (Fig. 1A1, A2). Fragments of superficially injured cartilage demonstrated very limited cell ingrowth framed by the thickness of the injured layers (Fig. 1B1, B2). However, deep cell migration and extended repopulation were observed in defective areas with a loss of GAG content. Ingrowth appearance was deeper and voluminous in the completely degraded matrix (Fig. 1C1, C2, and D1, D2, respectively).

**Fig. 2:**
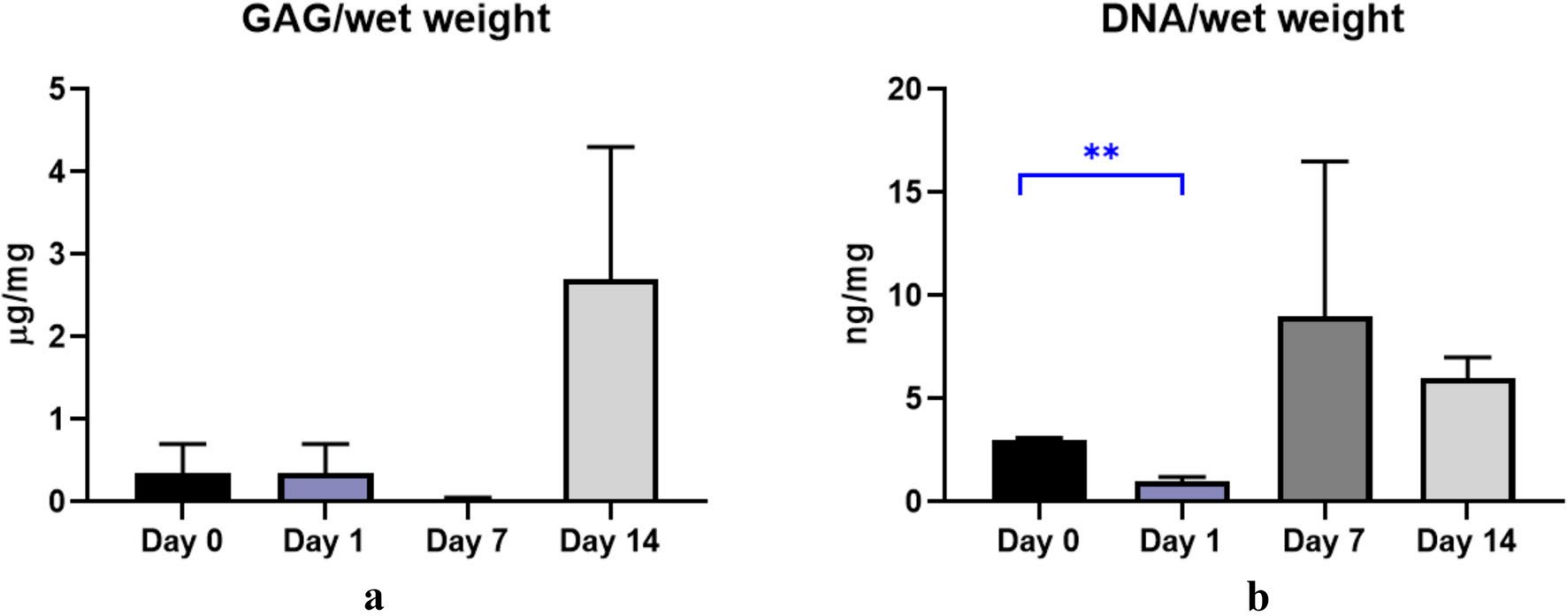
Concentration in 14 days: **A** GAG/wet weight, **B** DNA/wet weight. **p value < 0.01

The results of biochemical quantification of GAGs and DNA showed that by day 7, the amount of DNA increased significantly compared with day 1 and even exceeded the values in the original sample (Fig. 2). This indicates that chondrocytes are beginning to actively divide. At the same time, the quantitative values of GAG significantly decrease by day 7 (Fig. 2A), but sharply increase on day 14, when there is a decrease in the amount of DNA (Fig. 2B). This is explained by the fact that there is a decrease in cell division due to an increase in GAG synthesis de novo.

It is also worth noting that there were no statistically significant differences between GAG concentrations at 14 days.

### Regenerative effect of migrated chondrocytes on articular cartilage in vitro

**Fig. 3:**
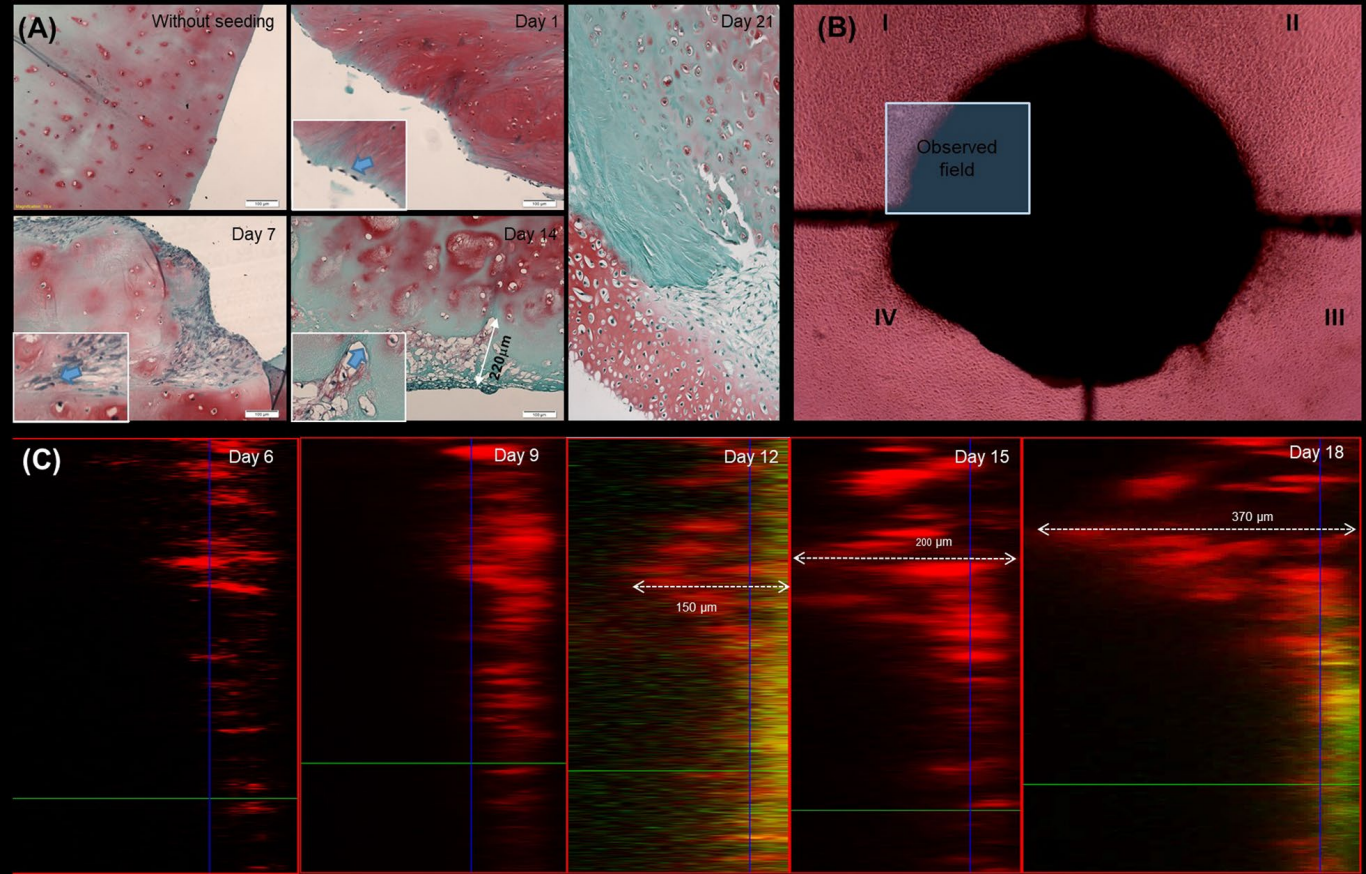
Assessment of chondrocyte migration dynamics during different cultivation periods: **A** fast-green, safranin-O, and hematoxylin histological staining of the cartilage matrix; **B** cartilage fragment co-cultured with nasal chondrocytes; **C** cell migration in a dynamic observation site, laser scanning electron microscopy

Nasal chondrocyte dynamics and migration inside the devitalized cartilage matrix by histological staining were assessed from day 0 to 21 as shown in Fig. 3A. Devitalized cartilage matrix without seeding was used as a control sample, a complete absence of cells can be seen. Adhesion of nasal chondrocytes and coverage of the cartilage matrix by a single cell monolayer were observed on the first day. On day 7 cartilage formation and migration of nasal chondrocytes into the matrix are already clearly visible. On day 14 of cultivation intercellular spaces are filled by cartilaginous matrix synthesized de novo with a considerable low amount of GAG (blue staining). Depth of cell penetration continues to increase up to 200 µm. On day 21 we also observed neo-formed cartilage rich in GAG (bright red/rose staining with safranin-O).

In order to investigate the behavior of hNCs and their guidance in altered cartilage we continuously observed the same area of cartilage tissue co-cultured with the cells. The fragment of cartilage co-cultured with hNCs and the area of dynamic observation (Fig. 3B). We applied PKH-26 membrane labeling to track the cells on the laser scanning confocal microscope. This allowed us to assess changes in the position of chondrocytes and cartilage repopulation in the same area.

hNCs migrated inside the cartilage matrix to a depth of 270 μm over a period of 18 days (Fig. 3C). The migration speed was found to be irregular: cells covered the last 150 μm approximately within the 6 days prior to the end of cell culture.

**Fig. 4:**
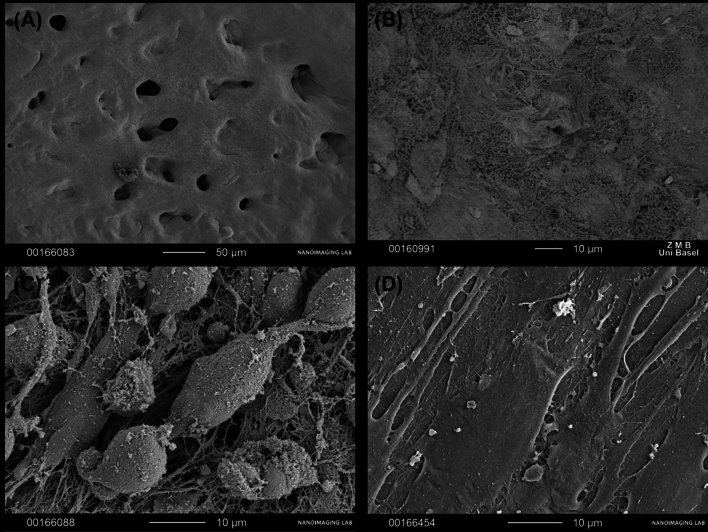
Scanning electron microscopy: **A** normal cartilage; **B** inflamed cartilage; **C** cartilage matrix populated with chondrocytes, day 7; **D** cartilage matrix populated with chondrocytes, day 21

To confirm the effect of cartilage regeneration using nasal chondrocytes, electron microscopy was performed as shown in Fig. 4. Figure 4A shows healthy, slippery and smooth cartilage, with no structural abnormalities. Whereas inflamed cartilage is loose and uneven, with intercellular fibers chaotically arranged, loose, and lacking structure (Fig. 4B).

After cell seeding on day 7, individual attached nasal chondrocyte cells are visible, and synthesis of intercellular substance is observed (Fig. 4C). At 21 days the regenerative effect of chondrocytes on the inflamed cartilage tissue can be observed: the smooth surface is now restored again and completely covered by neoformed cartilage with no gaps and spaces (Fig. 4D).

We cannot judge the migration of cells on the electron microscopy images.

## Discussion

Our preliminary findings provided further support for the hypothesis that human nasal chondrocytes are capable of interacting with the altered ECM of articular cartilage affected by arthritis. This interaction appears to be the underlying mechanism for regenerative repair in damaged cartilage. These basic findings also contribute to novel therapeutic approaches with nasal chondrocytes. Chondrocytes were previously characterized as cells highly sensitive to pro-inflammatory stimulation [35–37]. In particular, the appearance of inflammatory cytokines in the nearest cell environment is known to change their behavior. We seeded healthy and inflamed cartilage with nasal chondrocytes and found that the cells actively invade mainly pathologically altered cartilage. This effect is due to the fact that the cartilaginous matrix itself is capable of motivating chondrocytes for repopulation and cell ingrowth, despite the absence of synovial fluid, containing the majority of pro-inflammatory cytokines in the affected joints. hNCs migration and revitalization were found to be regulated by architectonics of ECM illustrating an endogenous mechanism for cartilage healing. In the same way, cells are restricted from cartilage penetration in healthy areas, which ensures the capability of the cells for specific guidance. In addition, it was found that the intensity of chondrocyte migration and their colonization of cartilage tissue correlates with the degree of its lesion with OA [37, 38]. That is, hNCs are capable of deep colonization migration into the cartilage affected by inflammation (penetration depth on day 18 reaches 370 μm). Cartilage recovery de nova is confirmed by electron microscopy images, where complete healing of cartilage surface occurs 3 weeks after cell seeding. We further confirmed the cartilaginous origin of neoformed tissue by the results of biochemical quantitative GAG analysis. The quantitative measurement of DNA indicated active cell proliferation. Our findings indicate that hNCs are responsible for the regeneration of cartilage affected by OA.

Currently, there were no clinical studies related to the changes of biomechanical properties of cartilage ECM after cell injections, as well as cell migration and seeding effectiveness. Therefore, it is worth emphasizing the importance of taking into account such parameters as proliferation rate and biochemical quantification of GAGs and DNA, which are indicators of knee articular cartilage matrix repair and show the predominant effectiveness of therapy with nasal chondrocytes over articular cartilage cells [19, 21, 39, 40]. Liu et al. demonstrated that hNCs, when incorporated into a hydrogel scaffold, can enhance cartilage differentiation and mechanical properties [41]. Similarly, Jeon et al. found that hNCs in 3D spheroids showed higher viability and chondrogenic capacity, leading to greater cartilage repair in a rat model [22]. Barthold et al. further enhanced this approach by developing a composite material of acellular ECM microparticles in a hydrogel, which promoted cell migration and integration while supporting native tissue architecture [42].

Human nasal chondrocytes (hNCs) are able to induce beneficial changes in such severely degenerated cartilage. Recent studies highlighted the effect of hNCs in treating advanced cartilage degeneration, particularly in osteoarthritis (OA). hNCs demonstrate superior performance in inflammatory environments compared to articular chondrocytes (ACs) and mesenchymal stromal cells (MSCs) [27]. They exhibit greater proliferation ability, chondrogenic capacity, and maintenance of chondrogenic properties after extensive culture expansion [22]. All those findings were confirmed by our study. Lim et al. (2020) [43] demonstrated that hNCs, when combined with collagen, significantly enhanced cartilage repair in rat models, showing greater growth rates and expression of chondrocyte-specific proteins compared with articular chondrocytes. Acevedo Rua et al. [28] confirmed these findings, demonstrating that nasal chondrocyte-based tissue-engineered cartilage (N-TEC) not only survived in vivo, but also positively influenced the inflammatory profile of OA joint cells. The use of N-TEC maintained its properties under inflammatory conditions and improved the condition of patients with advanced OA. Human nasal chondrocytes also outperform ACs and mesenchymal stromal cells in accumulating glycosaminoglycans (GAGs) and type II collagen under OA conditions [28]. It has also been noted that articular cartilage cells, which are used in traditional ACI procedures, have lower proliferative capacity and potential dedifferentiation during culture expansion, which may affect the quality of regenerated cartilage [44]. This dedifferentiation occurs rapidly in monolayer culture, affecting the cells’ ability to produce functional cartilage tissue [45]. In contrast, human nasal chondrocytes have a higher proliferative and chondrogenic capacity, resulting in sufficient cell mass for implantation, preservation of their phenotype, and manufacturing of the well-matured cartilage [19]. This suggests that hNC treatment may have broader applications beyond localized defects, potentially offering therapeutic benefits even in advanced stages of cartilage degeneration. However, there are still no data on the possibility of using human nasal chondrocytes in systemic inflammatory diseases, autoimmune diseases and similar disorders, which are contraindications for existing methods of cartilage regeneration, and this requires additional research.

It can be assumed that the loss of cartilage tissue due to arthritis and other inflammatory pathologies may be caused by pathological regulation of chondrocytes. In this case, cell therapy with autologous chondrocytes, harvested from ‘healthy’ tissue, would be an effective method to restore the natural microenvironment. Also, the observed effects of current cellular therapies using the donor allogeneic normal-regulated chondrocytes may be based on this effect. Thus, the application of donor nasal chondrocytes could be an effective tool for cartilage regeneration. However, the replacement of synovial fluid with the elimination of pro-inflammatory cytokines may be helpful during cell therapy, resulting in targeted cartilage healing. The manifestation of cell tropism on pathologically altered cartilage may be due to the endogenous cellular regulation of chondrocytes [46, 47]. Our findings may pave the way for novel approaches in cartilage tissue engineering in situ with immediate cell seeding in the operating room.

## Limitations

The study utilized a small sample size, limiting its representativeness of the broader population. Experiments were conducted on specimens exhibiting extensive degenerative changes obtained post-total knee arthroplasty, and effects on specimens with other types of cartilage pathologies have not been assessed. Notably, the capacity of human nasal cartilage chondrocytes to infiltrate and induce beneficial modifications in severely degenerated cartilage is remarkable, and this capability has not yet been validated through transcriptomic profiling.

## Conclusion

Our preliminary results showed that human nasal chondrocytes are capable of infiltrating the pathologically altered ECM of articular cartilage damaged by arthritis, thereby promoting its repair to a physiologically relevant state.

## Data Availability

Not applicable.

## Abbreviations

MSC: Mesenchymal stem cell
ACI: Autologous chondrocyte implantation
ECM: Extracellular matrix
hNC: Human nasal chondrocytes
PBS: Phosphate-buffered saline
FBS: Fetal bovine serum
sGAG: Glycosaminoglycan
DNA: Desoxyribonucleic acid
DMMB: Dimethylmethylene blue
OA: Osteoarthritis
N-TEC: Nasal chondrocyte-based tissue-engineered cartilage
