# Correction: Pathologically altered articular cartilage attracts intense chondrocyte invasion into the extracellular matrix: in vitro pilot study

**DOI:** 10.1186/s43019-024-00253-2

**Published:** 2025-01-20

**Authors:** Victoria A. Shestakova, Ilya D. Klabukov, Ilya V. Kolobaev, Longfeng Rao, Dmitry A. Atiakshin, Michael A. Ignatyuk, Mikhail E. Krasheninnikov, Bagavdin G. Ahmedov, Sergey A. Ivanov, Peter V. Shegay, Andrey D. Kaprin, Denis S. Baranovskii

**Affiliations:** 1 National Medical Research Radiological Center of the Ministry of Health of the Russian Federation, Koroleva st. 4, 249036 Obninsk, Russia; 2 Obninsk Institute for Nuclear Power Engineering of the National Research Nuclear University MEPhI, Obninsk, Russia; 3 Patrice Lumumba Peoples Friendship University of Russia (RUDN University), Moscow, Russia; 4 ETH Zurich, Zurich, Switzerland; 5 National Medical Research Center for Surgery named after A.V. Vishnevsky of the Ministry of Health of the Russian Federation, Moscow, Russia; 6 University of Basel, Basel, Switzerland; 7 FSBEI HE “Rosunimed” of MOH of Russia, Moscow, Russia

**Correction: Knee Surgery & Related Research (2024) 36:42** 10.1186/s43019-024-00249-y

Following publication of the original article [1], we have been notified that Funding statement was published incorrectly.

It is now as follows:

**Funding**

This research was partially supported by the Russian Science Foundation, Agreement No. 24-14-00393.

It should be as follows:

**Funding**

This research was partially supported by the Ministry of Science and Higher Education of the Russian Federation, Agreement No. 075-15-20-2021-1356 (15.SIN.21.0011, RF ID 0951.61321X0012).
